# Uncoordinated production of Laminin-5 chains in airways epithelium of allergic asthmatics

**DOI:** 10.1186/1465-9921-6-110

**Published:** 2005-09-22

**Authors:** Kawa Amin, Christer Janson, Lahja Sevéus, Kaoru Miyazaki, Ismo Virtanen, Per Venge

**Affiliations:** 1 Department of Medical Sciences, Clinical Chemistry, Uppsala University, Uppsala, Sweden; 2 Department of Medical Sciences, Respiratory Medicine and Allergology, Uppsala University, Uppsala, Sweden; 3 Division of Cell Biology, Kihara Institute for Biological Research, Yokohama City University, Yokohama, Japan; 4 Institute of Biomedicine/Anatomy, University of Helsinki, Helsinki, Finland

## Abstract

**Background:**

Laminins are a group of proteins largely responsible for the anchorage of cells to basement membranes. We hypothesized that altered Laminin chain production in the bronchial mucosa might explain the phenomenon of epithelial cell shedding in asthma. The aim was to characterize the presence of Laminin chains in the SEBM and epithelium in allergic and non-allergic asthmatics.

**Patients and methods:**

Biopsies were taken from the bronchi of 11 patients with allergic and 9 patients with non-allergic asthma and from 7 controls and stained with antibodies against the Laminin (ln) chains alpha1-alpha5, beta1-beta2 and gamma1-gamma2.

**Results:**

Lns-2,-5 and -10 were the main Laminins of SEBM. The layer of ln-10 was thicker in the two asthmatic groups while an increased thickness of lns-2 and -5 was only seen in allergic asthmatics. The ln gamma2-chain, which is only found in ln 5, was exclusively expressed in epithelial cells in association with epithelial injury and in the columnar epithelium of allergic asthmatics.

**Conclusion:**

The uncoordinated production of chains of ln-5 in allergic asthma could have a bearing on the poor epithelial cell anchorage in these patients.

## Background

Asthma is a chronic inflammatory disease of the lungs that may have allergic or non-allergic causes [1-3]. The allergic type of asthma is characterized by the accumulation of eosinophils, mast cells and lymphocytes of the Th2-type in the bronchial mucosa, whereas the non-allergic asthma has a substantial accumulation of neutrophils in addition to eosinophils and mast cells [3]. Structural changes and remodelling of the bronchial mucosa with signs of epithelial injury, subepithelial basement thickening, smooth muscle hypertrophy, increased vascularization and innervation are prominent features of the allergic type of asthma and less prominent in the non-allergic type [3].

Basement membranes (BMs) are built of cell-polymerizing networks of type IV collagens and laminins connected by nidogen/entactin [4,5]. The major role of laminin for epithelial cells is to anchor them to BM for cell differentiation and maintenance of cell function. Laminins are heterotrimeric molecules made up by one α, one β and one γ chain. Until today we know of five α-chains, three β-chains and three γ-chains. These chains combine into at least 14 different Laminins (lns) i.e. lns 1–14. The distribution of these Laminin isoforms varies between tissues, but in most BMs more than one Laminin is present. The chains of laminins have different regions that function by binding to cellular receptor molecules among which the most abundant are integrins, dystroglycan and the recently characterized Lutheran blood group antigen [4,6]. Several studies have shown the fundamental importance of intact Laminins in the BMs, since mutations may give rise to serious diseases such as epidermolysis bullosa in which the anchoring of the skin is grossly impaired [7]. Laminins also interact with many other cells and promote migration and angiogenesis and their functions in tumour invasion is one of the hot research topics of today [4].

The injury of the respiratory epithelium in the bronchi in allergic asthmatics may be one of the mechanisms underlying bronchial hyperresponsiveness which is one of the main features of asthma [8-11]. The mechanisms behind the fragility of the epithelium in allergic asthmatics, i.e. the propensity of the epithelium to shed from its anchorage to the subepithelial basement membrane (SEBM) and basal cells have not been explained. Since one obligatory component in this anchoring process is mediated by Laminins, we hypothesized that uncoordinated production of Laminin chains might contribute to weaken these anchoring forces. Our aim was therefore to describe the presence of the various Laminins in the epithelium and especially SEBM of allergic asthmatics in comparison with non-allergic asthmatics and healthy non-asthmatic controls.

## Materials and methods

### Subjects

Bronchial biopsies were collected from twenty-nine non-smoking adults divided into the following groups: healthy controls (n = 7), patients with allergic asthma (n = 11) and patients with non-allergic asthma (n = 9) (Table 1). All patients had a clinical asthma diagnosis, current asthma symptoms and increased responsiveness to inhaled methacholine. The allergic asthma patients all had a positive skin prick test (≥ 3 mm) for at least one common allergen (birch, timothy grass (*Phleum pratense*), mugwort (*Artemisia vulgaris*), cat, dog, horse, house dust mite (*Dermatophagoides pteronyssinus*), *Cladosporium*, and *Alternaria*.) while the non-allergic asthma patients and the controls all had a negative skin prick test. All patients with allergic asthma were examined outside the birch and grass pollen season (April to August).

**Table 1:** Patient characteristics (n or median (range))

	Healthy control (n = 7)	Allergic asthma (n = 11)	Non-allergic asthma (n = 9)
Age (yr)	25 (22–43)	37 (29–63)	41 (17–62)
Sex (M/F)	2/5	2/9	2/7
FEV_1 _(% pred)	98 (71–120)	94 (72–109)	86 (72–97)
FVC (% pred)	98 (78–109)	100 (86–118)	87 (76–96)
Symptom score *	0 (0–1)	2 (0–4)	2 (1–2)
PEF-variability (%)	5 (3–9)	11 (6–22)	10 (5–20)
PC_20 _(mg/ml)	-	2.7 (0.07–32)	8.7 (1.0–32)
Pollen allergy	0	9/11	0
Pet allergy	0	11/11	0
Mite allergy	0	4/11	0
Mould allergy	0	3/11	0

*number of symptoms recorded in a questionnaire during 2 weeks (9)

All but one allergic and one non-allergic patient with asthma were on regular treatment with inhaled glucocorticosteroids (budesonide 200–800 g/day) and inhaled β_2_-agonists as needed. The average use of inhaled glucocorticosteroids was similar in the two asthma groups. A more detailed description of the study population has been presented in a previous report[3].

### Bronchoscopy

The patients were given 10 mg diazepam (Stesolid^®^, Dumex, Copenhagen, Denmark) orally and 0.5 mg atropine (Atropin, NM Pharma, Stockholm, Sweden) subcutaneously 30 minutes before the investigation. The upper airways were anaesthetized with lidocain hydrochloride (Xylocain, Astra, Södertälje, Sweden). Using a flexible fibre bronchoscope (Olympus P 20D) with a FB 15C 2,0 mm forceps (Olympus), two biopsies were taken from each of three different airway levels in the right lung: (A) in the upper lobe bronchus immediately after the division from the main bronchus, (B) at the division between the middle and lover lobe bronchi and (C) from the main lower lobe divisions. The specimens were immediately examined under a light microscope to ensure the presence of a complete mucosa and fixed as described below. The patients were instructed to take their regular asthma sprays the morning of the bronchoscopy.

The study was conducted in accordance with the Declaration of Helsinki and was approved by the ethics committee at the Faculty of Medicine at the University of Uppsala.

### Immunohistochemistry

The expression of different Laminin chains in the epithelium and in the subepithelial basement membrane was studied in frozen sections by the use of monoclonal antibodies and the alkaline phosphate-anti-alkaline phosphatase method (APAAP) visualization system. Mouse monoclonal antibodies (MAbs) against the ln α1 chain (clone 161 EB7)[12], ln α4 chain (clone 168FC10)[13], ln α5 (clone 4C7) [14,15], ln β1 chain (clone 114DG.)[16], ln β2 chain (clone C4, obtained from the hybridoma C4 developed by Joshua Sanes obtained from the Developmental Studies hybridoma Bank developed under the auspices of the NICHD and maintained by the University of Iowa, Department of Biological Sciences, Iowa City, IA 52242), ln γ1 chain (113BC7), ln γ2 chain (clone D4B5) [17] were produced and characterized as described earlier. MAb against lns α2 and α3 chains (clones Lam M and P3H9-2, respectively) were purchased from Chemicon International, Temecula, California, USA.

The bronchial biopsy specimens were taken from the upper lobe and frozen immediately in melting propane previously cooled in liquid nitrogen and further processed as described in detail previously [3]. Incubation with antibodies to ln α1 chain (diluted 1:4 in PBS), ln α2 chain (diluted 1:500 in PBS), ln α3 chain (diluted 1:1000 in PBS) ln α4 chain (diluted 1:5 in PBS), ln α5 (diluted 1:300 in PBS), ln β1 chain (diluted 1:400 in PBS), ln β2 chain (diluted 1:750 in PBS), ln γ1 chain (diluted 1:400 in PBS) and ln γ2 chain (diluted 1:1000 in PBS) were performed at room temperature in a humid chamber for 20 h and terminated by two rinses in PBS. The bound antibodies were visualized with the alkaline phosphates-anti-alkaline phosphatase method (APAAP kit K670, Dakocytomation, Glostrup, Denmark) using fast red substrate. The sections were counterstained with Mayer's hematoxylin (Merck; Darmstadt, Germany) for two minutes and mounted with Faramount (S 3025, Dako). In the negative controls, the primary antibodies were omitted.

### Microscopic Evaluation of Sections

The Leica DMLB microscope (Wetzlar GmbH, Germany) was equipped with a Leica Microsystems digital camera (DC 300F) connected to a PC-computer. The images were captured and saved in the computer for further evaluation using the software package Qwin v2.7. In each biopsy two subsequent sections were evaluated. The thickness of the Laminin layers (in μm) was measured in immunolabeled frozen sections using a X10 objective and the computerized image analysis system after calibration with the aid of a stage micrometer. Measurements were carried out on 100 randomly selected sites per section and the results expressed as the mean of these measurements. The variation in estimation of structural changes between the two microscopic sections varied between 4–8% (% coefficient of variation). All slides were assessed by an observer blinded to the diagnosis of the patient.

### Statistics

All statistics were calculated using non-parametric tests. Comparisons between the three groups were initially performed by means of analysis of variance (Kruskal-Wallis test). In case of significance paired group comparisons were performed with the Mann-Whitney U-test. A p-value of <0.05 was regarded as statistically significant.

## Results

### Ln α-chain

**Figure 1:**
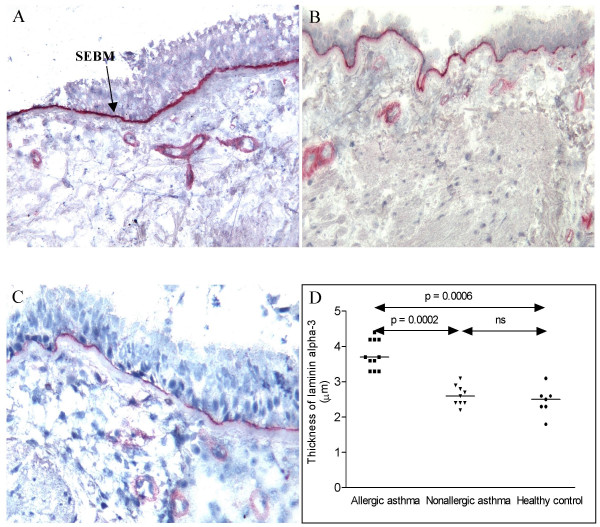
Cryostat sections of bronchial biopsies stained with antibodies against the ln α3 chain. Allergic asthma (A), non-allergic asthma (B) and healthy control (C) (original magnification ×170). The comparison of the thickness of SEBM is shown and the significant differences between the groups shown in the figure. Mayer's hematoxylin.

**Figure 2:**
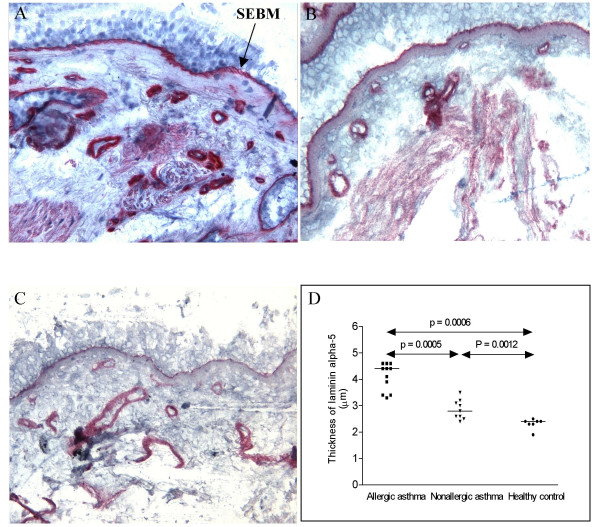
Cryostat sections of bronchial biopsies stained with antibodies against the ln α5 chain. Allergic asthma (A), non-allergic asthma (B) and healthy control (C) (original magnification ×170). The comparison of the thickness of SEBM is shown and the significant differences between the groups shown in the figure. Mayer's hematoxylin.

With MAb against ln α1-chain we saw some weak patchy staining of the SEBM in biopsies from patients with allergic asthma, but not in the biopsies of healthy subjects or of subjects with non-allergic asthma. Alfa2-chains were found in the SEBM and were significantly thicker in biopsies from allergic asthmatics as compared to non-allergic asthmatics and healthy controls (table 2). The α3-chain was also found in the SEBM and the layer was significantly thicker in biopsies from allergic asthmatics compared to non-allergic asthmatics and controls (Figure 1, table 2). No staining of the epithelium was discerned with the antibodies against the α3-chain. No staining of the SEBM or epithelium was found with antibodies against α4-chains. The staining of SEBM with antibodies against the α5-chain showed a thicker layer in the allergic than in the non-allergic asthmatics. The layer α5 was also thicker in the non-allergic asthmatics than in the controls (Figure 2).

**Table 2:** The thickness of various laminin chains in SEBM (μm)

**Laminin chain**	**Healthy controls**	**Allergic asthma**	**Non-allergic asthma**
α_1_-chain	No staining	Patchy, weak staining	No staining
α_2_-chain	1.94 (1.70–2.20)	2.83 (2.50–3.30)**, ‡‡‡	2.19 (1.8–2.90)
α_3_-chain	2.46 (1.80–3.10)	3.77 (3.30–4.40)***, ‡‡‡	2.61 (2.20–3.10)
α_4_-chain	No staining	No staining	No staining
α_5_-chain	2.31 (1.90–2.50)	4.10 (3.30–4.60)***, ‡‡‡	2.86 (2.40–3.50) **
β_1_-chain	2.13 (1.60–2.80)	4.84 (4.10–5.50)***, ‡‡‡	3.29 (3.10–3.80) ***
β_2_-chain	1.93 (1.60–2.60)	2.34 (2.10–2.800)*, ‡‡	2.04 (1.80–2.300)
γ_1_-chain	2.47 (1.90–3.00)	4.86 (4.20–5.40) ***, ‡‡‡	3.49 (2.90–3.90) ***
γ_2_-chain	2.03 (1.70–2.40)	2.96 (2.60–3.50)**, ‡‡‡	2.36 (2.00–2.80)

Results are given as medians and interquartile ranges. Differences between groups were calculated by the Mann-Whitney non-parametric test and statistical differences shown in the table. **, *** indicate p < 0.01 and p < 0.001, respectively, in the comparison between either of the two asthma groups with the results of the healthy controls. ‡, ‡‡, ‡‡‡ indicate p < 0.05, <0.01 and 0.001, respectively, in the comparison between the two asthma groups.

### Ln β-chains

Ln β1-chains were seen in the SEBM and the thickness was significantly higher in biopsies from allergic asthmatics as compared to non-allergics and healthy controls (Table 2). The staining of SEBM with antibodies against the β2-chain showed a slight increase of the thickness in allergic asthmatics as compared to non-allergic asthmatics and controls, but with no difference between non-allergic asthmatics and controls (Table 2).

### Ln γ-chains

**Figure 3:**
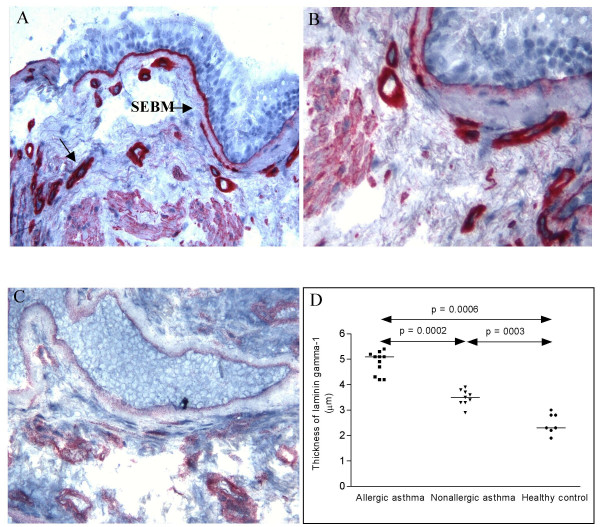
Cryostat sections of bronchial biopsies stained with antibodies against the ln γ1 chain. Allergic asthma (A), non-allergic asthma (B) and healthy control (C) (original magnification ×170). The comparison of the thickness of SEBM is shown and the significant differences between the groups shown in the figure. Mayer's hematoxylin.

**Figure 4:**
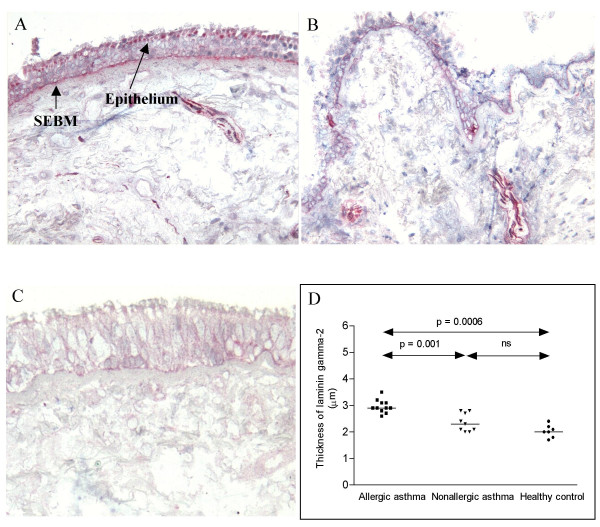
Cryostat sections of bronchial biopsies stained with antibodies against the ln γ2 chain. Allergic asthma (A), non-allergic asthma (B) and healthy control (C) (original magnification ×170). As shown in the figure epithelial staining was found both in the apical part of the columnar epithelium and in the basal cells. Staining of the apical part of the columnar epithelium was only found in intact epithelium from allergic asthmatics. The comparison of the thickness of SEBM is shown and the significant differences between the groups shown in the figure. Mayer's hematoxylin.

**Figure 5:**
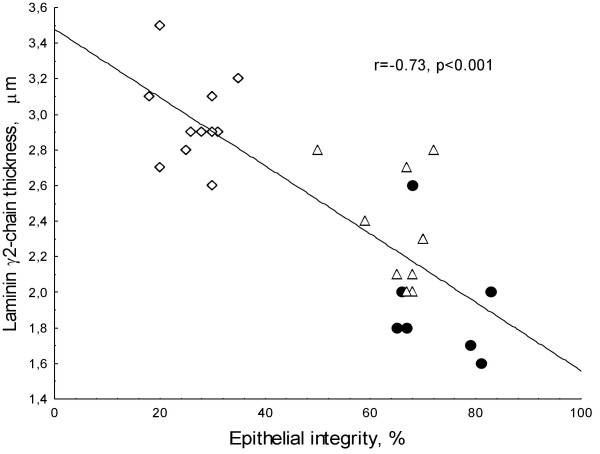
The relationship between epithelial integrity (%) in the bronchial mucosa and the thickness of the ln γ2-chain (μm). Overall there was a negative correlation between the epithelial integrity and the ln γ2-chain thickness (r = -0.73, p < 0.001), whereas no significant correlations were observed within the respective patient group. Diamonds represent allergic asthmatics, squares represent non-allergic asthmatics and closed circles healthy controls.

Staining of the biopsy with antibodies against the ln γ1-chain revealed an increased thickness in the SEBM in both allergic and non-allergic asthma as compared to controls and a much thicker layer in allergic asthmatics as compared to non-allergics (Figure 3). The staining of the bronchial mucosa with antibodies against the γ2-chain revealed a thicker layer in the SEBM in allergic asthmatics as compared to both non-allergic asthmatics and controls (Figure 4, Table 2). The antibodies also stained the epithelium. Thus, as shown in the figure the staining was found both in the apical part of the columnar epithelium and in the basal cells. Staining of the apical part of the columnar epithelium was only found in intact epithelium from allergic asthmatics, whereas staining of the basal cells was seen in all three groups in areas of epithelial injury. A close correlation was also found between the epithelial integrity in the three study groups and the thickness of the ln γ2-chain (figure 5).

When combining the information on individual laminin chains, we found that lns-2 (α2β1γ1),-5 (α3β3γ2) and -10 (α5β1γ1) were the main laminins of the SEBM. The layer of ln-10 was thicker in the two asthmatic groups while an increased thickness of lns-2 and -5 was only seen in allergic asthmatics. The staining of ln γ2-chain in the absence of ln α3 in epithelial cells does not fit with any presently known laminin.

## Discussion

This study has systematically investigated the presence of most Laminin chains in the epithelium and SEBM of both allergic and non-allergic asthmatics. The primary object was to test the hypothesis that differences in Laminin chain compositions in SEBM might help explain the phenomenon of epithelial fragility and shedding as is typically seen in biopsies of allergic asthmatics [3]. We found several alterations in the Laminin chain composition in the SEBM of allergic asthmatics. Most of these differences seemed quantitative rather than qualitative. Unexpectedly we found distinct qualitative differences with respect to ln-5 chain compositions that may have a bearing on the poorer anchorage of epithelial cells to BM in allergic asthma. The finding of a close correlation between ln γ2-chain deposition and epithelial injury indeed emphasises the close relationship between laminin chain production and epithelial injury as is observed in certain subjects with asthma. However, the relationship does not tell us whether epithelial shedding is a cause of the uncoordinated laminin chain production or whether the uncoordinated production is a consequence of the repair processes induced by epithelial injury.

The unexpected findings were both related to the ln γ2-chain staining. According to the present knowledge the γ2-chain is only found as part of ln-5 (α3β3γ2) [4,18]. In our biopsies we found exclusive staining with the antibodies against the γ2-chain in the epithelium, with no sign of simultaneous staining with antibodies against the α3-chain. The epithelium staining could be a reflection of the fact that airways epithelium is a producer of the ln γ2-chain and that the staining reflects the deposition of non-secreted protein. It was of particular interest that we found accumulation of immunoreactivity in the apical part of intact columnar epithelium in allergic asthmatics, but not in the other two groups, whereas staining of the basal cells were seen in all three groups in areas of epithelial destruction. This staining was observed without any concomitant staining of the complementary ln-5 α3 chain, which suggests an uncoordinated production of the γ2 chain in the epithelium of allergic asthmatics resulting in the intracellular accumulation of the γ2 chain, since the extracellular secretion probably requires the assembly of the heterotrimeric molecule [18]. The staining pattern could also indicate the presence of a hitherto unrecognised Laminin or alternatively that the α3 chain had been proteolytically modified with the loss of the particular epitope recognized by our monoclonal antibodies [19].

The intense staining of the cytoplasm of basal cells in areas of epithelial injury suggests that the basal cells are producers of the ln γ2 chain and probably also of the whole heterotrimeric complex of ln-5, although no staining of the other two chains was observed. Indeed, sole expression of ln γ2 chain has been shown in invading tumour cells[4,20], which shows that uncoordinated production of the three ln-5 chains may take place under certain conditions. It is also of interest that Lappi-Blanco et al. in a recent report found ln γ2 chain expression to be increased in regenerating epithelial cells and also found γ2 chain in basal cells of normal bronchus [21]. These results suggest that our findings of intense staining seen in the basal cells at areas of tissue injury may be a sign of re-epithelialization and repair.

The SEBM showed the presence of mainly three Laminins i.e. ln-5 (α3β3γ2), ln-10 (α5β1γ1) and ln-2 (α2β1γ1). The two former were expected based on earlier findings [4,22], whereas the presence of ln-2 mostly is associated with BMs surrounding tissues such as muscles and nerves [23]. In a previous report we indicated the wide presence of α1-chains in SEBM, which is seemingly contrasted by the present results[24]. Those results, however, were based on the false assumption that the monoclonal antibody 4C7 specifically recognizes α1-chains, which is not the case. The 4C7 antibody only recognizes the α5-chains [14,15]. As was previously found the thickness of the Laminin layer in the SEBM was increased in allergic asthmatics as compared to both non-allergic asthmatics or healthy controls [3]. This difference was most obvious for ln-10, since also the thickness found in non-allergic asthmatics was increased as compared to healthy non-asthmatic controls. This was contrasted by the increased thickness of ln-5 and -2, which was only seen in allergic asthmatics. These differences, therefore suggest qualitative differences in the production of various chains in allergic and non-allergic asthmatics, which may relate to the differences in the inflammatory processes going on in these two diseases. The allergic asthma being eosinophil-mast cell-Th2 driven and the non-allergic asthma being more neutrophil-mast cell driven, although eosinophils are also present at increased amounts in the non-allergic asthma [3].

As mentioned above the primary aim of this work was to test the hypothesis that an imbalance in Laminin chain production might explain the observed epithelial cell loss in allergic asthmatics. This hypothesis is seemingly refuted by our data, since others have shown that ln-5 induces the formation of hemidesmosomes [25], which actually promote stable cell:matrix adhesion. Another interesting property of ln-5 is the biological activity of the proteolytically modified fragments, which might modify cellular behaviours [26]. However, it should also be noted that mutations or modifications of any of the chains of ln-5 are associated with severe disease due to separation of epithelia from the underlying basement membrane [7]. Thus, we cannot exclude any processing of ln-5 in the inflamed tissue of allergic asthma as an explanation of poor anchorage of the epithelial cells in the bronchi to the underlying basement membrane.

## Conclusion

We conclude that Laminin chain deposition in the epithelium and SEBM of allergic and non-allergic asthmatics differs in qualitative and quantitative terms and that there is a close relationship between ln γ2-chain deposition and epithelial injury. The uncoordinated production of the chains of ln-5 in the epithelium of allergic asthmatics may be of particular interest, since ln-5 promotes the formation of hemidesmosomes, which promote stable cell matrix adhesion.

## Abbreviations

alkaline phosphate-anti-alkaline phosphatase (APAAP), basement membranes (BMs), Laminin (ln), Mouse monoclonal antibodies (Mabs), subepithelial basement membrane; (SEBM),

## Competing interests

The author(s) declare that they have no competing interests.

## Authors' contributions

Kawa Amin and Lahja Sevéus have done the immunohistochemistry part of the study and also been involved in the evaluation of the data

Christer Janson has been responsible for recruiting the patients and has been involved in the evaluation of the data

Per Venge initiated the study and has been the principal author of the paper

Ismo Virtanen and Kaoru Miyazaki have provided the unique antibodies and also been involved in the evaluation of the data

## References

[B1] Holgate ST (1999). The epidemic of allergy and asthma. Nature.

[B2] Busse WW, Rosenwasser LJ (2003). Mechanisms of asthma. J Allergy Clin Immunol.

[B3] Amin K, Ludviksdottir D, Janson C, Nettelbladt O, Bjornsson E, Roomans GM, Boman G, Seveus L, Venge P (2000). Inflammation and structural changes in the airways of patients with atopic and nonatopic asthma. Am J Respir Crit Care Med.

[B4] Patarroyo M, Tryggvason K, Virtanen I (2002). Laminin isoforms in tumor invasion, angiogenesis and metastasis. Semin Cancer Biol.

[B5] Libby RT, Champliaud MF, Claudepierre T, Xu Y, Gibbons EP, Koch M, Burgeson RE, Hunter DD, Brunken WJ (2000). Laminin expression in adult and developing retinae: evidence of two novel CNS laminins. J Neurosci.

[B6] Kikkawa Y, Moulson CL, Virtanen I, Miner JH (2002). Identification of the binding site for the Lutheran blood group glycoprotein on laminin alpha 5 through expression of chimeric laminin chains in vivo. J Biol Chem.

[B7] McGowan KA, Marinkovich MP (2000). Laminins and human disease. Microsc Res Tech.

[B8] Fireman P (2003). Understanding asthma pathophysiology. Allergy Asthma Proc.

[B9] Ludviksdottir D, Janson C, Bjornsson E, Stalenheim G, Boman G, Hedenstrom H, Venge P, Gudbjornsson B, Valtysdottir S (2000). Different airway responsiveness profiles in atopic asthma, nonatopic asthma, and Sjogren's syndrome. BHR Study Group. Bronchial hyperresponsiveness. Allergy.

[B10] Crimi E, Spanevello A, Neri M, Ind PW, Rossi GA, Brusasco V (1998). Dissociation between airway inflammation and airway hyperresponsiveness in allergic asthma. Am J Respir Crit Care Med.

[B11] Kushima A, Motojima S, Yamai T, Makino S (1990). The participation of epithelial desquamation in the increase of bronchial hyperresponsiveness after antigen challenge in patients with bronchial asthma. Allergy (Japan).

[B12] Virtanen I, Gullberg D, Rissanen J, Kivilaakso E, Kiviluoto T, Laitinen LA, Lehto VP, Ekblom P (2000). Laminin alpha1-chain shows a restricted distribution in epithelial basement membranes of fetal and adult human tissues. Exp Cell Res.

[B13] Petajaniemi N, Korhonen M, Kortesmaa J, Tryggvason K, Sekiguchi K, Fujiwara H, Sorokin L, Thornell LE, Wondimu Z, Assefa D, Patarroyo M, Virtanen I (2002). Localization of laminin alpha4-chain in developing and adult human tissues. J Histochem Cytochem.

[B14] Engvall E, Earwicker D, Haaparanta T, Ruoslahti E, Sanes JR (1990). Distribution and isolation of four laminin variants; tissue restricted distribution of heterotrimers assembled from five different subunits. Cell Regul.

[B15] Tiger CF, Champliaud MF, Pedrosa-Domellof F, Thornell LE, Ekblom P, Gullberg D (1997). Presence of laminin alpha5 chain and lack of laminin alpha1 chain during human muscle development and in muscular dystrophies. J Biol Chem.

[B16] Virtanen I, Lohi J, Tani T, Korhonen M, Burgeson RE, Lehto VP, Leivo I (1997). Distinct changes in the laminin composition of basement membranes in human seminiferous tubules during development and degeneration. Am J Pathol.

[B17] Mizushima H, Koshikawa N, Moriyama K, Takamura H, Nagashima Y, Hirahara F, Miyazaki K (1998). Wide distribution of laminin-5 gamma 2 chain in basement membranes of various human tissues. Horm Res.

[B18] Tunggal P, Smyth N, Paulsson M, Ott MC (2000). Laminins: structure and genetic regulation. Microsc Res Tech.

[B19] Hintermann E, Quaranta V (2004). Epithelial cell motility on laminin-5: regulation by matrix assembly, proteolysis, integrins and erbB receptors. Matrix Biol.

[B20] Katayama M, Sekiguchi K (2004). Laminin-5 in epithelial tumour invasion. J Mol Histol.

[B21] Lappi-Blanco E, Kaarteenaho-Wiik R, Salo S, Sormunen R, Maatta M, Autio-Harmainen H, Soini Y, Paakko P (2004). Laminin-5 gamma2 chain in cryptogenic organizing pneumonia and idiopathic pulmonary fibrosis. Am J Respir Crit Care Med.

[B22] Virtanen I, Laitinen A, Tani T, Paakko P, Laitinen LA, Burgeson RE, Lehto VP (1996). Differential expression of laminins and their integrin receptors in developing and adult human lung. Am J Respir Cell Mol Biol.

[B23] Patton BL (2000). Laminins of the neuromuscular system. Microsc Res Tech.

[B24] Altraja A, Laitinen A, Virtanen I, Kämpe M, Simonsson BG, Karlsson SE, Håkansson L, Venge P, Sillastu H, Laitinen LA (1996). Expression of laminins in the airways in various types of asthmatic patients: A morphometric study. Am J Respir Cell Mol Biol.

[B25] Michelson PH, Tigue M, Jones JC (2000). Human bronchial epithelial cells secrete laminin 5, express hemidesmosomal proteins, and assemble hemidesmosomes. J Histochem Cytochem.

[B26] Ghosh S, Stack MS (2000). Proteolytic modification of laminins: functional consequences. Microsc Res Tech.

